# Use of Selective Serotonin Reuptake Inhibitors and Risks of Stroke in Patients with Obsessive Compulsive Disorder: A Population-Based Study

**DOI:** 10.1371/journal.pone.0162239

**Published:** 2016-09-09

**Authors:** Che-Sheng Chu, Po-Han Chou, Ching-Heng Lin, Chin Cheng, Chia-Jui Tsai, Tsuo-Hung Lan, Min-Wei Huang, Gerald Nestadt

**Affiliations:** 1 Department of Psychiatry, Puli Branch, Taichung Veterans General Hospital, Nantou, Taiwan; 2 Department of Psychiatry, Taichung Veterans General Hospital, Taichung, Taiwan; 3 Department of Psychiatry, School of Medicine, National Yang Ming University, Taipei, Taiwan; 4 Department of Photonics, National Chiao Tung University, Hsinchu, Taiwan; 5 College of Biological Science and Technology, National Chiao Tung University, Hsinchu, Taiwan; 6 Department of Medical Research, Taichung Veterans General Hospital, Taichung, Taiwan; 7 Department of Psychiatry, China Medical University Hospital, Taichung, Taiwan; 8 Institute of Clinical Medicine, National Yang-Ming University, Taipei, Taiwan; 9 Center for Neuropsychiatric Research, NHRI, Miaoli, Taiwan; 10 Department of Psychiatry, Chiayi Branch, Taichung Veterans General Hospital, Chiayi, Taiwan; 11 Department of Psychiatry and Behavioral Sciences, School of Medicine, Johns Hopkins University, Baltimore, Maryland, United States of America; Istituto Di Ricerche Farmacologiche Mario Negri, ITALY

## Abstract

**Background:**

Previous research has suggested a link between antidepressants use and the development of cerebrovascular events, but there has never been any study investigating the risk of stroke in obsessive-compulsive disorder (OCD) patients treated with a selective serotonin reuptake inhibitor (SSRI).

**Methods:**

A retrospective observational cohort study was conducted using data from the National Health Insurance Database of Taiwan between the year of 2001 and 2009. A total of 527 OCD patients with 412 subjects in the SSRI use group and 115 in the non SSRI use group were included. Multivariable Cox proportional-hazards models were used to explore the associations between SSRI use and the occurrence of stroke, controlling for age, gender, concomitant medications, and comorbid medical illnesses.

**Results:**

A total of nineteen OCD patients were diagnosed with new onset of stroke during the follow-up period including six cases in the SSRI group and thirteen in the non SSRI use group. SSRI use was demonstrated to be associated with a decreased risk of stroke (hazard ratio [HR] = 0.30; 95% confidence interval [CI] = 0.10–0.86, P = 0.02). The increase in age-related risk of strokes was 2.55 per decade (HR = 2.55; 95% CI = 1.74–3.75, P<0.001). Alternatively, sex, concomitant use of aspirin and non-steroidal anti-inflammatory drugs, and comorbidities with angina pectoris, diabetes mellitus, hypertension, and hyperlipidemia were not found to be associated with an increased risk for stroke in OCD patients.

**Conclusions:**

Our study showed that SSRI use was associated with decreased risk of stroke in OCD patients. Further investigation into the possible biological mechanisms underlying the relationship between stroke and SSRI use in OCD patients is warranted.

## Introduction

Obsessive-compulsive disorder (OCD) is a chronic psychiatric illness characterized by repetitive overt and covert acts which can be obsessions (i.e., intrusive recurrent thoughts), compulsions (i.e., repetitive behaviors in response to obsessions), or both [1]. The lifetime prevalence of OCD is 2.3% in the United States [2] and 0.7% in Taiwan [3]. Symptoms of OCD are associated with reduced quality of life [4] and cause significant functional impairments [5]. The World Health Organization has ranked OCD as one of the 10 most handicapping conditions by lost income and decreased quality of life [6]. Current evidence supports the use of selective serotonin reuptake inhibitors (SSRIs) as first-line pharmacotherapy for treatment of OCD, for its’ effectiveness and safety [7]. The U.S. food and drug administration (FDA) has approved several SSRIs such as fluoxetine, fluvoxamine, paroxetine, and sertraline for treatment of OCD.

Recently, several studies have investigated the associations between antidepressants and cerebrovascular events but the results have been inconsistent [8–17]. While most studies did not find an association [8,9,11,12,14,16], more recent studies have revealed that SSRI use might be associated with increased risk of ischemic stroke [10], hemorrhagic stroke [15], or both [13, 17]. In a case-crossover study in 2011, Wu and colleagues reported increased risks of strokes among SSRI users with short-term exposure (14 days) based on a large population-based analysis in Taiwan [17]. More recently, Hung et al. also demonstrated increased risk of strokes among geriatric population prescribed with at least 2 months of SSRIs, using the national health insurance research database (NHIRD) in Taiwan [13]. However, an important methodological issue for these observational studies is that the risk of stroke among SSRI users may be confounded by indication for prescription. Besides psychiatric illness such as depression and anxiety disorders, antidepressants are widely used for treatment of various chronic and neuropathic pain disorders, such as fibromyalgia, migraine, and for reducing the pain of diabetic neuropathy [18]. Therefore, we do not know how much of the increase in stroke risk is attributable to the medication rather than the underlying treated diseases or other associated risk factors that distinguish users and non-users [19]. For example, previous studies have demonstrated that illnesses depression [20], panic disorder [21], and migraine [22] have an increased risk of stroke compared with the general population. Therefore, it is crucial for clinical physicians to understand the risk of stroke associated with SSRI use in each specific patient population like OCD.

However, to the best of our knowledge, there has never been any study investigating the risk of stroke in OCD patients treated with SSRIs. The National Health Insurance (NHI) program of Taiwan covers most of the population, and most medical institutions in Taiwan, and is therefore one of the largest insurance databases in the world. The NHIRD contains all claims from ambulatory and inpatient care, and has provided valuable information for a variety of epidemiological studies [23–27]. Therefore, the aim of this study was to examine the risk of stroke in OCD patients treated with SSRIs using the NHIRD in Taiwan.

## Methods

### Data sources

Our study was based on the patient data from NHIRD in Taiwan. The Taiwan NHIRD is a claims database maintained by the Department of Health and the National Health Research Institutes of Taiwan. The NHI program was launched in Taiwan on March 1^st^ 1995. By the end of 2010, more than 23.07 million of Taiwan’s 23.16 million people were enrolled in the program [28]. Starting in 1999, the Bureau of NHI (NHIB) began to release all claims data in electronic format to the public under the NHIRD project. The database provides scrambled patient identification numbers, dates of birth, gender, and diagnosis in the format of the International Classification of Diseases, Ninth Revision, Clinical Modification (ICD-9-CM), medication prescribed, medical costs, medical care facilities, and their specialties. In the present study, the medical data for a total of 1,000,000 persons (approximately 4% of Taiwan’s population), were randomly selected from the Taiwan NHIRD for the analysis. The NHIB uses a systematic sampling method to randomly extract a representative database from the entire database. There are no statistically significant differences in age, sex, and medical costs between the sample group and all other enrollees. We included medical data from individuals who were enrolled between January 1^st^, 2000, and December 31^st^, 2009, except those with questionable or inadequate basic data.

### Ethics statement

The Institutional Review Board of Taichung Veterans General Hospital approved this study (IRB No. CE13151). NHIB released the dataset which consists of de-identified secondary data for research purposes, and therefore written consent from the study subjects was not obtained. The Institutional Review Board of Taichung Veterans General Hospital issued a formal written waiver for consent for this study.

### Study design

We conducted a retrospective observational study on the impact of SSRIs use on the incidence of stroke in patients diagnosed with OCD in Taiwan from 2001 to 2009. Patients were excluded if before 2001: 1. Ages are younger than twenty years; 2. Ever diagnosed with a stroke (ICD-9-CM codes 430.XX to 438.XX); 3. Hospitalized or received ambulatory treatment for any mental disorder (ICD-9-CM code: 290.XX-319.XX). This ensured that subsequent diagnoses of stroke and OCD were of newly onset. Study subjects were identified from 2001 to 2007 and the observation period to identify first stroke events or death extended until December 31, 2009 to ensure that study subjects could be followed for at least 3 years.

### Study participants

New cases of OCD were ascertained by the diagnosis of ICD-9-CM code 300.3. To ensure the validity of diagnosis, at least 2 consensus OCD diagnoses after the index medical visit was required. To exclude the possible confounding effects of other psychiatric disorders, patients with any mental disorder (ICD-9-CM codes: 290.XX-319.XX) one year prior to the index medical visit for OCD diagnosis were excluded.

### Definition of SSRIs exposure

In the present study, we focused on the long-term SSRI use and the incidence of stroke. Medication use was defined by receiving a prescription for a SSRI (paroxetine, fluoxetine, sertraline, citalopram, escitalopram, or fluvoxamine) for at least two consecutive months during January 1, 2001, to December 31, 2007. This criteria was the same us those adopted in our previous studies [13]. Patients who used only 1-month SSRI were excluded from the analysis because high possibilities of irregular SSRI use; the others were defined as non SSRI exposure group. The final sample in the study included 527 OCD patients, 412 subjects in the SSRI use group and 115 in the non SSRI use group.

### Definition of incidence of stroke

Participants were met criteria for incident stroke if they received at least two consecutive diagnoses of codes (430.XX to 438.XX) according to ICD-9-CM during the observation period after the index medical care visit for OCD.

### Statistical analysis

Distributions in age, gender, concomitant prescribed medications related to strokes (aspirin and Non-steroidal Anti-inflammatory Drugs [NSAIDs]) and comorbid medical conditions ((angina pectoris, diabetes mellitus (DM), hyperlipidemia, hypertension, atrial fibrillation, and myocardial infarction)) were examined by using χ^2^-tests. Multivariable Cox proportional-hazards models were used to explore the associations between SSRI use and risk of stroke, controlling for age, gender, concomitant medications, and medical comorbidities. All statistical tests were two sided, conducted at significance level of 0.05, and reported using *P* value and 95% confidence intervals (CIs). All analyses were performed using the SAS software, version 9.2 (SAS Institute, Cary, NC).

## Results

A total of nineteen participants had new onset of stroke during the follow-up period: six cases were in the SSRI use group and thirteen in the non SSRI use group. Table 1 compares the distribution of demographic characteristics, concomitant medications related to strokes, and relevant comorbid medical disorders between SSRI and non SSRI use groups. No instances of myocardial infarction or atrial fibrillation were observed in either group. In comparison to the SSRI use group, significantly more participants in the non SSRI use group were diagnosed with DM (*P* < 0.05) and hypertension (*P* < 0.02). In addition, the SSRI exposure group tended to be significantly younger than non SSRI exposure group.

**Table 1:** Demographic and clinical characteristics of OCD subjects (n = 527).

Variables	Non SSRI exposure group (n = 115)		SSRI exposure group (n = 412)		*P* value for χ^2^ test
	n	(%)	n	(%)	
Age (year)					
20–29	17	14.78	121	29.37	<0.001
30–39	25	21.74	142	34.47	
40–49	27	23.48	73	17.72	
50–59	29	25.22	54	13.11	
60–69	9	7.83	16	3.88	
≥70	8	6.96	6	1.46	
Sex					
Female	63	54.78	224	54.37	0.9372
Male	52	45.22	188	45.63	
Aspirin					
No	112	97.39	398	96.6	0.6718
Yes	3	2.61	14	3.4	
NSAID					
No	29	25.22	142	34.47	0.0611
Yes	86	74.78	270	65.53	
Angina Pectoris					
No	114	99.13	405	98.3	0.5201
Yes	1	0.87	7	1.7	
DM					
No	108	93.91	402	97.57	<0.05
Yes	7	6.09	10	2.43	
Hyperlipidemia					
No	105	91.3	388	94.17	0.2679
Yes	10	8.7	24	5.83	
Hypertension					
No	101	87.83	388	94.17	0.02
Yes	14	12.17	24	5.83	
Stroke					
No	102	88.7	406	98.54	<0.0001
Yes	13	11.3	6	1.46	

AP, angina pectoris; NSAID, Nonsteroidal Anti-inflammatory Drugs; DM, diabetes mellitus.

As shown in Table 2, after adjusting for other confounding factors with multivariable Cox proportional-hazards model, SSRI use was demonstrated to be associated with a significantly decreased risk of stroke (Hazard Ratio [HR] = 0.30; 95% CI = 0.10–0.86, *P* = 0.02). In addition, the increase in age-related risk of strokes was 2.55 per decade (HR = 2.55; 95% CI = 1.74–3.75, *P* < 0.001). Furthermore, sex, concomitant use of aspirin and NSAIDs, and comorbidity with angina pectoris, DM, hypertension, and hyperlipidemia did not show to increase the risk for stroke in OCD patients.

**Table 2:** The cox proportion hazard regression model of stroke in OCD (n = 527).

Variable*	Hazard Ratio	95% CI		*P* value
		Lower	Upper	
SSRI (Use/ Non-use)	0.302	0.106	0.863	0.0254
Age (every 10 years)	2.558	1.742	3.755	<0.0001
Sex (Male/ Female)	1.423	0.55	3.684	0.4668
AP (Yes/ No)	2.621	0.274	25.047	0.4027
NSAID (Yes/ No)	4.04	0.872	18.717	0.0743
DM (Yes/ No)	2.262	0.497	10.307	0.2913
Hypertension (Yes/ No)	0.554	0.149	2.053	0.3765
Hyperlipidemia (Yes/ No)	2.384	0.626	9.082	0.2031

AP, angina pectoris; NSAID, Nonsteroidal Anti-inflammatory Drugs; DM, diabetes mellitus.

*Due to small numbers of patients who take aspirin, it was not included in the analysis

## Discussion

To the best of our knowledge, this is the first study to investigate the risk of stroke in OCD patients treated with SSRIs. In the present study, we find SSRI use was associated with a reduced risk of stroke in OCD patients, based on a nationwide health insurance database.

### OCD and risk of stroke

There has been an increasing body of evidence suggesting that anxiety disorders are an independent predictor of adverse cardiovascular events. However, the evidence linking anxiety disorders and strokes are far from compelling. Moreover, there has been little research examining the physical health specifically in OCD patients until recently. Boschen and his colleagues found that in comparison to patients with other psychiatric diagnoses admitted to an acute psychiatric unit, patients OCD were more likely to have elevated serum cholesterol and serum creatinine levels [29]. Albert et al also found that the prevalence of the metabolic syndrome (21.2%) was higher in OCD patients compared to the general population in Italy [30]. These risk factors, an elevated serum creatinine [31,32], hyperlipidemia [33], and the metabolic syndrome [34,35] are known to be associated with an increased rate of strokes; it seems plausible that these risk factors should affect OCD patients to the same extent as others.

### Duration of SSRIs exposure and risk of stroke

Our results demonstrated that SSRI use was associated with reduced risk of stroke in OCD patients, which was inconsistent with those reported in recent studies. There are several possible explanations. First, it has been proposed that antidepressants may have acute detrimental influence but chronic beneficial effects on stroke risk [17]. The risks of stroke are probably higher in patient who newly uses antidepressants. Therefore, risk of stroke with antidepressant use may be varied by duration of treatment. For example, Chen and colleagues reported that antidepressant may increase risk of ischemic stroke only among current users (antidepressant within 30 days before stroke). A prospective cohort study by Smoller et al. also showed the antidepressant increased the risk of hemorrhagic stroke among new users of antidepressant [15]. However, it is still unclear whether the association between short-term antidepressant use and stroke is truly related to drug exposure or to acute phase of the treated underlying diseases, which might make patients more vulnerable to cardiovascular events. Second, SSRI use might express different efficacy pattern from patients of other psychiatric disorders in OCD patients. For example, Bloch et al reported the dose-response relationship of SSRI in OCD, showing that the greater side effect burden caused by higher doses of SSRI will be counterbalanced by the greater treatment efficacy [36], while Bollini et al showed this treatment pattern could not find in major depressive disorder (MDD) [37]. Therefore, discrepancies between our results and these of prior studies may be due to differences in methodology (long treatment duration) consideration and characteristics of study samples.

### SSRIs use and decreased risks of stroke

Platelets play an important role in thrombus formation and in the repair of vascular injury [38,39]. Serotonin concentration in platelets is crucial in maintaining the homeostatic function of platelets and the serotonin transporter (5-HTT) carrier protein functions to maintain the homeostasis of platelet serotonin concentration and serotonin level in blood [40]. Several weeks of treatment with a SSRI has been shown to inhibit 5-HTT and to block the reuptake of serotonin, leading to depletion of serotonin storage in platelets [41,42]. Additionally, both *in vitro* and *ex vivo* studies showed that SSRIs decreased adenosine diphosphate (ADP) or collagen induced platelet aggregation [43,44]. These effects may lead to attenuation of platelet activation and decrease the risk of thromboembolism formation [45]. This point was supported by several previous studies. In a population-based case-control analysis, Schlienger et al. provided evidence that SSRI could decrease risk for acute myocardial infarction [46] and several studies demonstrated no elevated risk of ischemic stroke under SSRIs use [8] or even some neuroprotective effect of SSRIs were reported [47]. Although SSRIs had the possibility of increased hemorrhage, the absolute risks between SSRI use and increases risk of hemorrhagic stroke are likely to be very low based on a meta-analysis report [48]. Therefore, it is plausible to consider that anti-platelet effects of SSRIs by reducing thromboemoblism could decrease the risk of strokes in OCD patients. The underlying mechanisms for the protective effects of SSRIs in OCD warrants further investigation.

### Physical comorbidities and risk of stroke

We did not find DM, hypertension, or hyperlipidemia associated with increased risks of stroke, which is inconsistent with previous studies [33,49]. There are several possible explanations for this finding. First, it may suggest that OCD patients represent a special group in whom DM, hypertension, or hyperlipidemia is not associated with the risk of stroke. A population based mortality study showed that there was a lower risk of death early in life for persons with OCD, although it was higher later in life; this may be consistent with reduced health risk in OCD [50]. Second, it is probably due to relatively small numbers of incident cases of these physical comorbidities could be included for statistical analysis. Therefore, small numbers of cases may make the regression model un-robust, as we could see from the rather wide range of confidence intervals of these 3 diseases (Table 2).

### Strength and limitations

The major strength of this study is that the findings were based on an analysis of a large sample from a nationwide population-based database, which minimized selection bias. In addition, this is the first study to investigate the SSRI-associated risk of stroke in OCD patients. However, our results should be viewed in the light of several limitations. First, relatively few incidence cases of stroke in OCD patients (n = 19) were identified, which reduces the power of this study. This was a result of the conservative definition for new-onset stroke; persons with a history of stroke prior to 2000 were excluded, i.e. recurrent episodes of stroke were excluded. In addition, we cannot examine the associations between SSRI and different types of strokes (i.e., ischemic or hemorrhagic stroke) due to limited incident cases of stroke. To solve the problem, a larger database should be used. The Taiwan NHIRD released only a random sample of the entire database for research use, preventing a larger sample size for this study. Second, OCD diagnoses, which rely on administrative claims data reported by hospitals, may be less accurate than diagnoses made according to standardized instruments (e.g., Structural Clinical Interview for DSM-IV Axis I Disorders). To ensure the validity of the diagnoses, all of the study subjects had at least two consensus OCD diagnoses after the index ambulatory care visit. In addition, the NHIB randomly samples a fixed percentage of claims from every hospital each year to ensure diagnostic validity through reviewing the symptomatologies documented in the medical records by an independent group of professional experts, and false claims would be fined [21]. Furthermore, some may question the accuracy of stroke-related coding in the NHIRD; however, previous studies, including one using NHIRD, have examined the validity of stroke diagnoses in claims data and showed high positive predictive values [51,52]. Third, some important risk factors for stroke, such as cigarette smoking, alcohol consumption, obesity, and body mass index, were not available in the NHIRD. Fourth, it was not possible for us to assess medication adherence from the NHIRD. Fifth, strokes may have been diagnosed long before the registry of NHIRD was established 1995, or undiagnosed prior to the start of study; this could not be detected in, or excluded from this study. Finally, since this study was conducted in Chinese population, generalization of these findings to different populations outside Taiwan is limited.

## Conclusion

Although previous studies have found an association between cerebrovascular events and antidepressant use, we did not find an increased risk of stroke in OCD patients treated with SSRIs. Furthermore, our study showed that SSRI use was associated with decreased risk of stroke in OCD patients. Further investigation into the possible biological mechanisms underlying the relationship between stroke and antidepressants use in OCD patients is indicated.
